# A Measles Epidemic Threshold in a Highly Vaccinated Population

**DOI:** 10.1371/journal.pmed.0020316

**Published:** 2005-10-18

**Authors:** Jacco Wallinga, Janneke C. M Heijne, Mirjam Kretzschmar

**Affiliations:** **1**Centre for Infectious Disease Epidemiology, National Institute for Public Health and the Environment, Bilthoven, the Netherlands; Health Protection AgencyUnited Kingdom

## Abstract

**Background:**

Mass vaccination against measles has successfully lowered the incidence of the disease and has changed the epidemic pattern from a roughly biennial cycle to an irregular sequence of outbreaks. A possible explanation for this sequence of outbreaks is that the vaccinated population is protected by solid herd immunity. If so, we would expect to see the fraction of susceptible individuals remaining below an epidemic threshold. An alternative explanation is the occurrence of occasional localised lapses in herd immunity that allow for major outbreaks in areas with a low vaccine coverage. In that case, we would expect the fraction of susceptible individuals to exceed an epidemic threshold before outbreaks occur. These two explanations for the irregular sequence of measles outbreaks can be tested against observations of both the fraction of susceptible individuals and infection attack rates.

**Methods and Findings:**

We have estimated both the fraction of susceptible individuals at the start of each epidemic year and the infection attack rates for each epidemic year in the Netherlands over a 28-y period. During this period the vaccine coverage averaged 93%, and there was no sustained measles transmission. Several measles outbreaks occurred in communities with low vaccine coverage, and these ended without intervention. We show that there is a clear threshold value for the fraction of susceptible individuals, below which only minor outbreaks occurred, and above which both minor and major outbreaks occurred. A precise, quantitative relationship exists between the fraction of susceptible individuals in excess of this threshold and the infection attack rate during the major outbreaks.

**Conclusion:**

In populations with a high but heterogeneous vaccine coverage, measles transmission can be interrupted without establishing solid herd immunity. When infection is reintroduced, a major outbreak can occur in the communities with low vaccine coverage. During such a major outbreak, each additional susceptible individual in excess of the threshold is associated with almost two additional infections. This quantitative relationship offers potential for anticipating both the likelihood and size of future major outbreaks when measles transmission has been interrupted.

## Abstract 2

High vaccine coverage does not necessarily protect against major outbreaks of measles in unvaccinated individuals in areas where vaccine coverage is low.

## Introduction

Measles is one of the most contagious diseases of humans, and an important cause of childhood deaths. The global effort to reduce measles mortality aims to achieve routine measles vaccination coverage of at least 90% in every district throughout the world. So far, this effort has resulted in a dramatic decline in deaths from measles [1]. The high vaccination coverages have changed the epidemic pattern from a roughly biennial cycle to an irregular sequence of outbreaks [2–4]. A proper understanding of the size and timing of these outbreaks is a prerequisite for adequate monitoring of a vaccination programme, and essential for assessing the risk of future measles outbreaks.

The size of an outbreak depends on both the fraction of susceptible individuals in the population and on chance events in the transmission process [5]. The fraction of susceptible individuals determines the expected size of an outbreak. Already in 1927, Kermack and McKendrick predicted that there should exist a critical threshold level for the fraction of susceptible individuals below which introduction of infection can only lead to minor outbreaks [6]. This so-called threshold theorem [7] underlies the concept of herd immunity, and it explains why it is possible to eradicate an infectious agent even without achieving complete vaccine coverage [8,9]. Variability in the size of outbreaks arises due to chance events in the transmission process. This variability becomes very large when the fraction of susceptible individuals is close to the epidemic threshold level [4,10]. When the fraction of susceptible individuals exceeds the threshold level, chance events determine whether a minor or a major outbreak will occur. The probability that the outbreak will be a major one increases with the fraction of susceptible individuals in excess of the threshold [5,11]. In their 1927 paper, Kermack and McKendrick showed that when the proportion of susceptible individuals is only slightly above the threshold level, almost two infections occur per susceptible individual in excess of the threshold level during a major outbreak [6]; this so-called second threshold theorem has been useful in calculating expected outbreak sizes [7,12]. Thus, the epidemic threshold for fraction of susceptible individuals marks a bifurcation in expected infection attack rates from only minor to both minor and major outbreaks, where the infection attack rate during major outbreaks is almost twice the excess fraction of susceptible individuals (Figure 1A).

**Figure 1:**
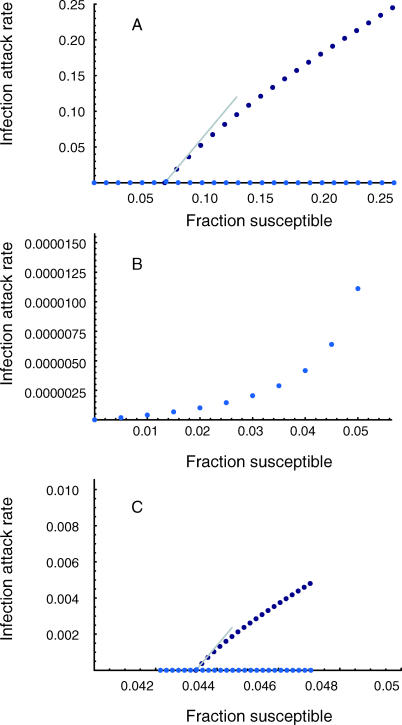
The Epidemic Threshold Concept (A) Expected infection attack rates during measles outbreaks in an idealised homogeneously mixing population. (B) Expected infection attack rates during measles outbreaks in a population protected by solid herd immunity. (C) Expected infection attack rates during measles outbreaks in a heterogeneous population consisting of a small community embedded in a larger population. The expectations are calculated for a stochastic “susceptible–exposed–infectious–recovered” (SEIR) model, with measles basic reproduction number *R*_0_ = 17 [2], and ten imported measles cases per year. The population structure is as reported for the Netherlands, where a small community of 300 000 persons with a variable fraction of susceptible individuals exists in a larger population of 15 million persons with a fraction of susceptible individuals of 0.043 [21]. Dark blue markers correspond to major outbreaks; light blue markers correspond to minor outbreaks. The solid gray line indicates the approximation, near the threshold, of the infection attack rate: two infections occur per susceptible individual in excess of the threshold.

Most observational studies on outbreak sizes in highly vaccinated populations have relied on two assumptions: first, the fraction of susceptible individuals is uniform throughout the population, and, second, the fraction of susceptible individuals remains below the epidemic threshold [3,4,13]. If these assumptions are met, the population is protected by solid herd immunity and no major outbreaks can occur. A simple one-to-one relationship exists between the observed infection attack rate and the fraction of susceptible individuals in the population (Figure 1B). This one-to-one relationship makes it possible to derive several epidemic variables of interest from observed outbreak sizes [3,4,10]. For example, countries and regions with elimination strategies for measles have been advised to monitor the average outbreak size as an indicator of “elimination status” [14]: smaller outbreaks are indicative of fewer susceptible individuals in a homogeneous population that is protected by solid herd immunity.

Many countries and regions have a heterogeneous distribution of the fraction of susceptible individuals [15]. This heterogeneity can be due to religious communities that refrain from vaccination (for example, the Amish in the United States of America [16]) or due to different vaccination programmes (for example, failure to implement additional vaccinations in the São Paulo region of Brazil [17]). The threshold concept, although often illustrated for an idealised homogeneous population, also applies with a few minor modifications to a heterogeneous population. In a simple heterogeneous population, consisting of a community with many susceptible individuals embedded in a population with few susceptible individuals, one still recognises an epidemic threshold for the average fraction of susceptible individuals in the entire population. And when a major outbreak hits the community with many susceptible individuals, approximately two cases occur per susceptible individual in excess of this population average threshold (Figure 1C). As a consequence, there is no longer a simple one-to-one relationship between the observed infection attack rate and the fraction of susceptible individuals in the population.

To our knowledge, there are no direct observations that show the precise nature of the relation between the fraction of susceptible individuals and infection attack rate during the irregular measles outbreaks that are characteristic for highly vaccinated populations. However, such observations could shed light on how one should interpret observed infection attack rates, and how to assess the risk of future measles outbreaks once endemic transmission has been interrupted. To overcome this deficiency, we have investigated the relation between the fraction of susceptible individuals and the infection attack rate for measles outbreaks over a 28-y period in the Dutch population. The Dutch data are ideally suited for an ecological study of measles outbreaks in a real, heterogeneous population: endemic measles transmission during this period was frequently interrupted in between the measles outbreaks, while the vaccination coverages and birth rates remained almost constant [18,19].

## Methods

### Study Population and Data Sources

Measles notification and vaccination began in 1976 in the Netherlands. From 1976 to 1987 a plain live attenuated measles vaccine was administered at the age of 14 mo. Since 1987, two doses of a combined measles, mumps, and rubella vaccine have been recommended at age 14 mo and 9 y. The vaccine coverage in the Netherlands has remained above 90% since 1978 (average coverage at 14 mo was 93%, range 91%–96%; data obtained from the Dutch Medical Inspectorate of Health). However, some municipalities reported vaccination coverages near 50%, owing to a high percentage of residents who refrained from vaccination on religious grounds. The size of the religious community in the Netherlands that refrains from vaccination is estimated at 300 000 persons, approximately 2% of the overall population [20]. The total population size increased from 13.7 million in 1976 to 16.3 million in 2004; the annual number of births varied between a lowest value of 170 000, recorded in 1983, and a highest value of 207 000, recorded in 2000 (data obtained from Statistics Netherlands, http://statline.cbs.nl/statweb). From 1976 to 2004, a total of 12 407 measles cases were notified; 96% of the measles cases that were notified from 1988 to 2004 were not vaccinated, mostly for religious and other ideological reasons (data obtained from the Dutch Medical Inspectorate of Health and from the National Surveillance System). Since 1976, two serological surveillance studies have been conducted in the Netherlands. In 1987, serum was obtained from an age-stratified sample of 2 041 inhabitants of the town of Nijmegen, and of these a fraction of 0.053 (standard error 0.015) tested negative for measles-specific antibodies; in 1996, serum was obtained from an age-stratified sample of 8 303 individuals from the Dutch population, and of these a fraction of 0.043 (standard error 0.002) tested negative for measles-specific antibodies [21].

### Data Analysis

We chose to measure time in “epidemic years” that started on 1 September and ended on 31 August. Around this time of the year the measles incidence was low, and often the transmission chain was interrupted. The various data sources provide information on a period that encompasses 28 such epidemic years, starting 1 September 1976 and ending 31 August 2004.

The infection attack rate was defined as the fraction of the population that became infected during an epidemic year. We estimated the number of measles infections per epidemic year as the number of reported cases divided by the reporting rates; the procedure for estimating these reporting rates is described below. The uncertainty introduced into the estimated infection attack rates due to incomplete reporting was small (for example, the 95% bootstrap interval for infection attack rate in 1976 was 72 × 10^−4^ to 78 × 10^−4^; the 95% bootstrap interval for infection attack rate in 2002 was 1 × 10^−6^ to 8 × 10^−6^).

Following the balance equation approach used by Fine and Clarkson [22] and Finkenstädt and Grenfell [23], we estimated the number of susceptible individuals by taking the number of susceptible individuals in the previous year, adding the recruitment of new susceptible individuals by birth over the epidemic year (after waning of maternal antibodies), and subtracting the depletion of susceptible individuals over the epidemic year by vaccination (using an estimated vaccine efficacy of 97%), infection, or death. We estimated the number of susceptible individuals at the start of the mass vaccination, together with reporting rates, by a weighted least-squares fit of the fraction of susceptible individuals to the observations from the serological surveys in 1987 and 1996; adjustments, in which updated estimates of the fraction of susceptible individuals were used, were made until convergence was reached [19]. The estimated fraction of susceptible individuals at the start of the mass vaccination was 0.05, which is within the theoretical expected range of 0.04–0.07 for a population in endemic equilibrium (assuming that the measles basic reproduction number is within the range 15–25; cf. [2,8]).

We estimated the measles reporting rates by a weighted least-squares fit of the fraction of susceptible individuals to the observations from the serological surveys in 1987 and 1996; the weighting factors were based on the reported standard errors for the results from these surveys [19]. We estimated that a fraction 0.024 of all measles infections were reported over the years 1976–1980, and we estimated that this reporting rate increased to a fraction 0.067 over the 1999–2000 epidemic year. These estimates were reassuringly close to other, independent estimates of the measles reporting rates in the Netherlands: 0.028 over the years 1976–1980 (van den Hof et al., cited in [19]) and 0.09 over the 1999–2000 epidemic year for a population served by a large general-practice group [24]. The derivation of realistic estimates for the reporting rates suggests that processes other than birth, vaccination, infection, or death—such as, for example, immigration or hypothesized waning of vaccine-induced immunity [25]—have contributed little to the number of susceptible individuals in the population.

Having reconstructed for each epidemic year a proportion of susceptible individuals and an infection attack rate, we classified epidemic years as either a minor or a major outbreak. We separated the epidemic years into two clusters by fitting two bivariate normal distributions. The distribution with the lowest mean infection attack rate represented a cluster of minor outbreaks, and the distribution with the highest mean infection attack rate represented a cluster of major outbreaks. We classified each epidemic year according to its relative likelihood of belonging to the cluster of major outbreaks. Epidemic years with a relative likelihood below 0.5 were classified as minor outbreaks, and epidemic years with a relative likelihood of 0.5 or higher were classified as major outbreaks. The relative likelihood of belonging to the cluster of major outbreaks was used as a weight for the subsequent regression analysis.

To determine the value of a threshold, if present, we used a weighted linear regression of infection attack rate on fraction of susceptible individuals. The intercept of the regression line with the axis for fraction of susceptible individuals gave the threshold value. The slope of the regression line gave the number of additional cases for each additional susceptible individual in excess of the threshold value.

## Results

We compared the time series of the fraction of susceptible individuals (Figure 2A) with the time series of infection attack rates (Figure 2B). Major outbreaks occurred when the fraction of susceptible individuals was relatively high at the start of the epidemic year. When major outbreaks were absent, the fraction of susceptible individuals steadily increased. Statistical analysis of the fraction of susceptible individuals and the attack rates revealed a bifurcating relation with a clear threshold level for the fraction of susceptible individuals near 0.043 (Figure 2C). Below the threshold level only minor outbreaks occurred. The infection attack rates during minor outbreaks tended to increase when the fraction of susceptible individuals was closer to the threshold level. Above this threshold level both minor and major outbreaks occurred. The likelihood of a major outbreak increased sharply with the fraction of susceptible individuals in excess of the threshold level. The size of a major outbreak also increased with the fraction of susceptible individuals in excess of the threshold level. During a major outbreak, the number of cases per excess susceptible individual was 1.61 (95% confidence interval 1.43–1.79).

**Figure 2:**
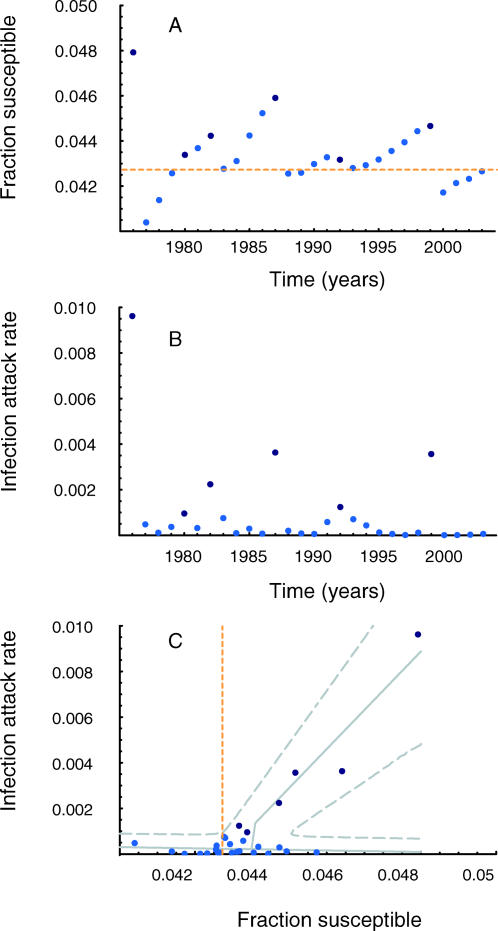
Observed Measles Outbreaks in the Netherlands, from 1 September 1976 to 31 August 2004 (A) The fraction of individuals susceptible to measles on 1 September of each year. (B) Infection attack rate of measles during each epidemic year from 1 September to 31 August. (C) Relation between fraction of susceptible individuals and attack rate. Dark blue markers correspond to major outbreaks; light blue markers correspond to minor outbreaks. Solid gray lines indicate the maximum likelihood values for infection attack rate; broken gray lines indicate the likelihood support region. The dotted yellow line in (A) and (C) indicates the threshold value for susceptible individuals below which solid herd immunity is achieved.

## Discussion

The irregular sequence of measles outbreak sizes reveals a clear epidemic threshold level: major outbreaks occur only if the fraction of susceptible individuals in the entire population is above this threshold level. To our knowledge, this is the most detailed observation in a human population of such a bifurcation from only minor towards both minor and major outbreaks.

The observations (Figure 2C) agree remarkably well with theoretical predictions for measles outbreaks in a heterogeneous population (see Figure 1C). The observed outbreaks at the population level are suggestive of an underlying heterogeneity, which is in accordance with the observation that most measles cases during the outbreaks were reported in communities with a low vaccine coverage [18,20]. The agreement between observations and theoretical predictions also suggests that the observed increase of infection attack rate with fraction of susceptible individuals may hold for other populations as well. Earlier studies have already recognised that clusters of persons refraining from vaccination may interfere with elimination of the disease [18] and pose an increased risk to others who comply with vaccination [26]. Our findings make precise why refusal of measles vaccine is a public health threat: one additional person who refrains from vaccination is associated with more than one—almost two—infected persons in subsequent major outbreaks.

We propose that the irregularities in size and timing of measles outbreaks in highly vaccinated populations can be understood within the familiar concept of the epidemic threshold. Previous approaches have relied on a statistical description of outbreak sizes, and derived their justification from a solid herd immunity in a homogeneous population [3,4]. In contrast, the estimated time series of susceptible individuals and attack rates, taken together, indicate that herd immunity may show occasional, localised lapses that allow for major outbreaks within communities with low vaccine coverage. The threshold concept reveals how the observed infection attack rates reflect the dynamics in the fraction of susceptible individuals, even when vaccination coverage is distributed variably throughout the population.

Great care should be taken in interpreting a time series of measles case notifications in the absence of a corresponding time series of the fraction of susceptible individuals. The occurrence of only a few measles cases over a long period of several years does not necessarily signal that solid herd immunity is achieved. It may alternatively signal an increasing risk of a future major measles outbreak. The picture of the bifurcating attack rates (see Figure 2C) helps to understand why. For example, during the years 2000–2003, few measles cases were reported, because the fraction of susceptible individuals remained below the epidemic threshold level and only minor outbreaks were reported. Since 2003, the threshold level has been exceeded. Only minor outbreaks have occurred because the probability of a major outbreak has remained small. However, if the fraction of susceptible individuals continues to increase over time as it did in previous years (see Figure 2A), both the probability of a major outbreak and the expected size of such an outbreak will increase over time. A major outbreak can be averted only if additional vaccination programmes target the communities at risk, or if import of measles cases into those communities is prevented. The information on both case notifications and the fraction of susceptible individuals can be further analysed by a mathematical model of transmission, or a data-driven model, to evaluate the effectiveness of alternative intervention strategies [8,12,23].

Once the current global effort to reduce measles mortality has achieved sustainable high measles vaccine coverage, many more countries and regions will experience irregular measles outbreaks. Our findings have important implications with respect to the design of disease-surveillance systems in populations with high vaccination coverages. First, it would be wise to strengthen the existing surveillance systems by estimating or monitoring the changes in the fraction of susceptible individuals over time, and by conducting serological surveys as part of that endeavour. Second, whenever the transmission chain has been interrupted, the estimated change in the fraction of susceptible individuals can be used to assess the risk of future major measles outbreaks.

**Patient Summary**

**Background**

Measles is a highly infectious, unpleasant disease that can cause death, especially in people who have low immunity or who are ill from other causes. For many years vaccination has been available, and in most Western populations there is a high coverage of vaccination; despite this vaccination outbreaks of measles do occur.

**Why Was This Study Done?**

The authors wanted to understand why outbreaks of measles occur in vaccinated populations, particularly how the rates of vaccination affected the chances of an outbreak occurring, and what the effect of people being unvaccinated was.

**What Did the Researchers Do and Find?**

They looked at vaccination records between 1 September 1976 and 31 August 2004 in the Netherlands, a highly vaccinated population for which coverage has remained above 90% since 1978. They found that between 1976 and 2004, a total of 12 407 measles cases were reported; 96% of the measles cases that were reported from 1988 to 2004 were in people who were not vaccinated (mostly for religious and other ideological reasons). Over a number of years they compared the number of people who could potentially get measles with the number who actually did. They found that only when the number of people who could potentially get measles exceeded a certain level was there a chance of a major outbreak, and they showed that one additional person who refrains from vaccination is associated with more than one—almost two—infected persons in subsequent major outbreaks.

**What Do These Findings Mean?**

It has long been known that vaccination rates should be kept high. This paper attempts to answer the questions, when is “high” high enough, and how bad is it if it is a little lower? The paper shows that one additional person who refrains from vaccination is associated with almost two infected persons in subsequent major outbreaks. The authors also suggest how surveillance can be enhanced to detect whether the rates of vaccination are high enough and suggest that there should be monitoring of the proportion of susceptible people and that health officials should ensure that this proportion should not increase. Refusal of measles vaccine could be considered a public health threat because of the effects of an unvaccinated individual on the rest of the population—that is, the chance of the epidemic spreading.

**Where Can I Get More Information Online?**

MedlinePlus has information on measles and the measles vaccine:

http://www.nlm.nih.gov/medlineplus/measles.html

The US Centers for Disease Control and Prevention has a Web page on the history of measles, with links to other information:

http://www.cdc.gov/nip/diseases/measles/history.htm

The World Health Organization has information on measles including in developing countries:

http://www.who.int/topics/measles/en/
